# Protective immunity against *Schistosoma japonicum* infection can be provided by IgG antibodies towards periodate-sensitive or periodate-resistant glycans

**DOI:** 10.1186/s13071-015-0842-1

**Published:** 2015-04-18

**Authors:** Wenci Gong, Fengjuan Huang, Yilei Ma, Hongmei Bai, Lan Yin, Jun Li, Chunxia Chen, Xindong Xu, Xiao-Ping Chen

**Affiliations:** Department of Immunology, Tongji University School of Medicine, Shanghai, China; Institute for Infectious Diseases and Vaccine Development, Tongji University School of Medicine, Shanghai, China

**Keywords:** Glycan, SjEA, *Schistosoma japonicum*, Monoclonal antibodies, Protective immunity

## Abstract

**Background:**

It has been well accepted that glycans present in schistosomes are highly antigenic. However, it is not clear what kind of worm glycans can affect the infected host to mount IgG responses and whether mounted anti-glycan IgG responses are protective.

**Methods:**

The contribution of antigenicity by glycans was measured by using competitive ELISA assay in sera from infected mice and humans. Monoclonal antibodies towards soluble *Schistosoma japonicum* egg antigens (SjEA) were generated from SjEA immunizated mice. The expression of glycans on surfaces of cercaria or young worm and their distributions were examined by immunofluorescence assay. The protective roles of glycans-specific mAbs were assayed by determination of the worm and egg burden in infected mice.

**Results:**

Both periodate-resistant glycans and periodate-sensitive glycans are antigenic in schistosome infections. When monoclonal antibodies against either periodate-sensitive or periodate-resistant glycans were administered prior to schistosome infections in mice, both kinds of anti-glycan antibodies were found to successfully provide protective immunity to infected mice.

**Conclusions:**

Both periodate-resistant and periodate-sensitive glycans are antigenic, and dominant anti-glycan IgG responses can play important roles in protective immunity in schistosome infected hosts.

**Electronic supplementary material:**

The online version of this article (doi:10.1186/s13071-015-0842-1) contains supplementary material, which is available to authorized users.

## Background

Schistosomiasis ranks as one of the most important health problems in developing countries due to global distribution and parasitic nature of the disease-causing pathogen schistosome [1]. Current treatments rely primarily on single chemotherapy by praziquantel (PZQ), but high rate of post-treatment reinfection, inability of PZQ treatment to interrupt transmission and possible occurrence of drug resistance all limit the use of chemotherapy as preventive measures [2]. As a result, development of effective and safe anti-schistosome vaccines is highly needed to prevent schistosome infection and to reduce transmission [1].

It has long been established that immunization with ionizing radiation-attenuated cercariae (RA) provides so far the best protective immunity in vaccinated rodents and primates [3,4]. However, this type of vaccine is not quite feasible for human use due to its complexity and potential side effects. Attempts have been made to use vaccines derived from recombinant proteins generated by prokaryotic expression. In contrast to the high efficacy seen in RA, few successes have been achieved from recombinant vaccine molecules trialed in mice as well as in large animals, regardless of whether vaccine molecules are designed toward *Schistosoma mansoni*, the species prevalent in Africa, or toward *Schistosoma japonicum*, the species prevalent in Asia [5,6]. One of the underlying causes attributing to different efficacies between RA and recombinant proteins could be due to a lack of epitopes important for induction of protective immunity when vaccines are generated by prokaryotic expressing system.

Possible protective epitopes lacked in such polypeptide vaccines can be glycans which are generated via glycosylation on proteins or lipids known to heavily occur on schistosome [7]. Both egg secretions and cercarial secretions of *Schistosoma mansoni* are found to have high mannose type, truncated type, complex and hybrid type of N-glycan structures, as well as mucin-type and novel type of O-glycan structures [8]. It is not known whether these abundantly expressed glycans by worms are simply a disguise to escape host immune surveillance as proposed [9,10], or some of these glycans are in fact involved in induction of protective immune responses. If the latter is true, glycan epitope is certainly needed to be considered in anti-schistosome vaccines.

IgG antibodies (IgGs) generated from infection by *Schistosoma mansoni* during both early- and egg- stage of infection are largely directed to glycans because markedly reduced IgGs binding activities are observed on periodate-treated schistosome antigens compared to untreated antigens [9,10]. This notion is largely based on the assumption that periodate treatment will alter most of glycan-associated epitopes attached to polypeptides or lipids, thus periodate-treated glycosylated antigens are conventionally named as deglycosylated antigens. The legitimacy of regarding periodate-treated antigens as deglycosylated molecules has been questioned [11]. Moreover, it is not clear if dominant anti-glycan IgG responses found in schistosome infection are associated with protective immunity.

In this report, we verified that IgGs in sera of *Schistosoma japonicum* infected mice and patients are predominantly targeted to untreated egg antigens (SjEA) with significantly reduced reactivities against periodate-treated egg antigens (pSjEA). In contrast to conventional assumption that periodate treatment will destroy all glycan-associated eiptopes, our data demonstrated that pSjEA still contained periodate-resistant sugars by lectin blotting. More importantly, the remained IgGs binding activities against pSjEA present in sera from infected patients and infected mice were primarily targeted to glycans but not to proteins by competitive inhibition ELISA assay. Therefore, dominant anti-glycan IgG responses induced in schistosome infection should not be explained that IgGs binding activities on periodate-treated schistosome antigens markedly reduced compared to untreated antigens as previous studies have revealed [9,10]. In fact, both periodate-sensitive and periodate-resistant glycans are prominently involved in donating antigenicity in infected host. More significantly, monoclonal antibodies towards both kinds of glycans were able to provide protective responses against worm infection. Results generated from this study provide evidence on the necessity to include glycans in selection of vaccine molecules to combat infections caused by pathogens like schistosome.

## Methods

### Ethics statement

All animal experiments were performed in strict accordance with the Regulations for the Administration of Affairs Concerning Experimental Animals (approved by the State Council of People’s Republic of China) and the Guide for the Care and Use of Laboratory Animals (Experimental Animal Center, Tongji University, certificated by Shanghai Committee of Science and Technology). All procedures performed on animals in this study were approved by the Committee on the Ethics of Animal Experiments of Tongji University (Permit Number: TJLAC-009-031).

### Mice and parasites

6**–**8 weeks old female Balb/c mice were purchased from SLAC laboratory (Shanghai, China). All mice were maintained under specific pathogen-free conditions and fed with standard laboratory food and water. Gender and age-matched mice were infected percutaneously with 30 ± 2 cercariae of *Schistosoma japonicum*, which were shed from infected *Oncomelania hupensis* snails provided by the National Institute of Parasitic Diseases at Shanghai, China. Young worms were recovered from hepatic portal vein of infected mice perfused by cold PBS pumped into the aorta artery 18 days post-infection. Both cercariae and worms will be used in immunofluorescence assay.

### Preparation and treatment of soluble egg antigens from *Schistosoma japonicum*

Soluble *Schistosoma japonicum* egg antigens (SjEA) were prepared as described with modifications [12]. Purified eggs were crushed with ultrasonication for 10s each run with interval break of 10s for 30 min in cold PBS. After being centrifuged at 13,000 rpm for 30 min, the supernatants were filtered through 0.22 μm filters and stored at-80°C until use.

**Figure 1:**
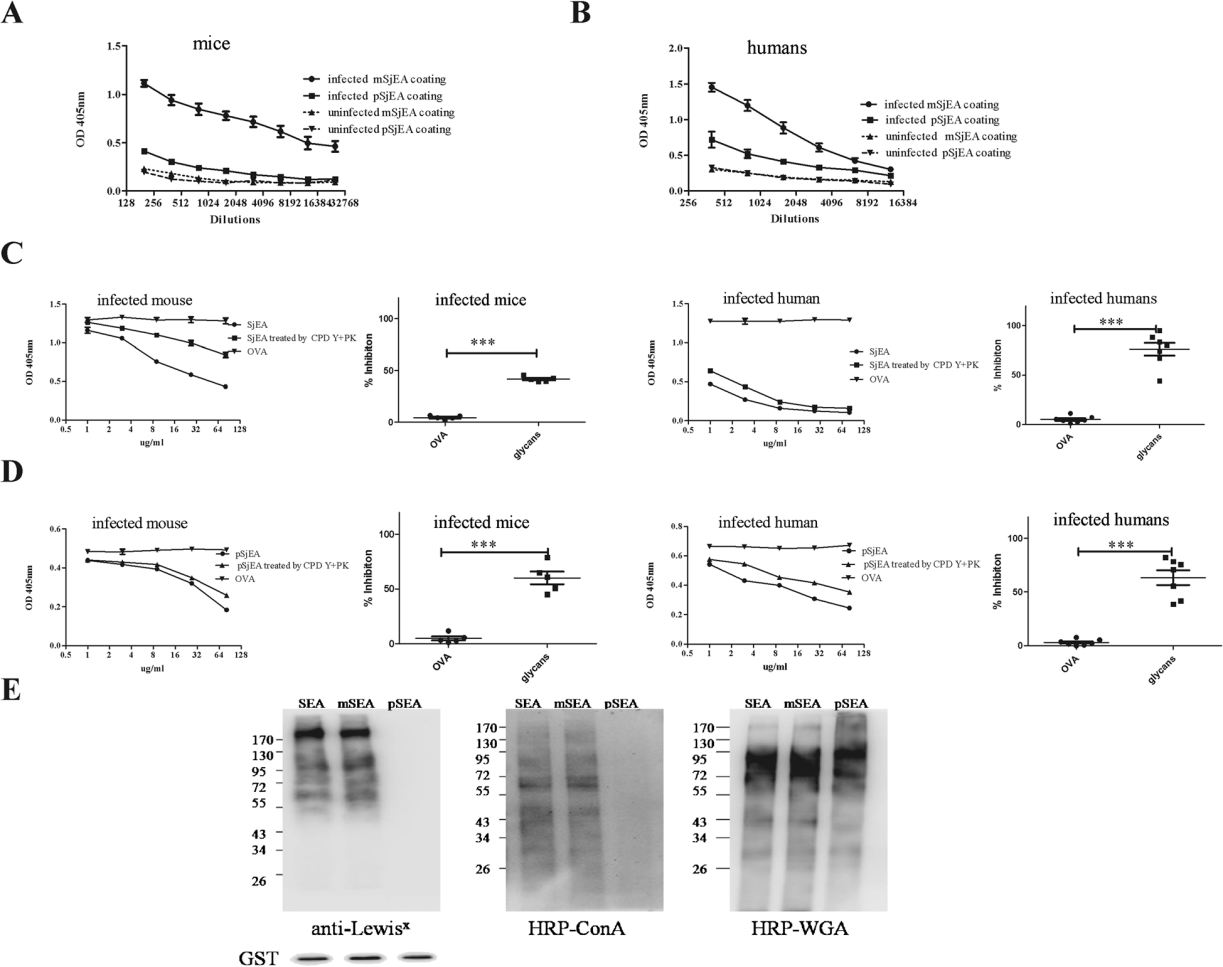
Inhibitory effects on IgGs binding activities by egg antigens devoid of proteins. **A** and **B.** Higher levels of IgG against untreated egg antigens (SjEA) than against periodate-treated SjEA (pSjEA) were found in sera from *Schistosoma japonicum* infected mice **(A)** or infected humans **(B)**. Data shown were mean ± SEM of OD values of variously diluted sera in infected mice (n = 5) or in infected patients (n = 7). Data on mice were representative of two separate experiments. **C.** SjEA devoid of proteins by proteinase K (PK) and carboxypeptidase Y (CPD Y) treatment was capable of inhibiting IgG bindings with SjEA whether in infected mice or infected humans at dose-dependent fashion. A representative competitive inhibition curve on IgGs in infected sera was shown on panel 1 (mouse) and panel 3 (human) using untreated SjEA as positive control for inhibition assay and OVA as negative control. A summary on calculated percentage of inhibition from infected mice (n = 5) or infected humans (n = 7) by proteinases-treated SjEA was shown at panel 2 and panel 4. **D.** pSjEA devoid of proteins by proteinases treatment was also capable of inhibiting IgG bindings with pSjEA at dose-dependent fashion. A representative competitive inhibition curve on IgGs in infected sera was shown on panel 1 (mouse) and panel 3 (human) using untreated pSjEA as positive control for inhibition assay and OVA as negative control. A summary on calculated percentage of inhibition from infected mice (n = 5) or infected humans (n = 7) by enzyme-treated pSjEA was shown at panel 2 and panel 4. **E.** Con A, WGA and anti**-**Lewis^x^ antibody staining of mock- and periodate-treated SjEA. Anti-GST was used as loading control. *t* test was used to analyze the significance of percent inhibition with *** as p < 0.001.

Periodate oxidation was performed as described [13]. 10 mM sodium metaperiodate (Sigma) was added to SjEA (2 mg/ml) dissolved in 50 mM sodium acetate buffer (pH 4.6) for 45 min. Oxidation reaction was stopped by adding sodium borohydride with final concentration at 50 mM. Mock-treated SjEA was subjected to the same procedure with omission of sodium metaperiodate treatment. After reaction, mock- or periodate-treated SjEA was extensively dialyzed against PBS and the concentration was readjusted based on protein concentration measured by Bradford method (Bio-Rad). The effects of metaperiodate treatment on glycans were examined by HRP-ConA, HRP-WGA and anti-Lewis^x^ staining as shown in Figure 1.

To release N-glycans from protein backbone, SjEA was enzymatically digested with PNGase F (NEB) as instructed by manufacturer’s protocol. Briefly, SjEA was denatured by heating at 100°C for 10 min in glycoprotein denaturing buffer (NEB). After cooling, PNGase F was used to remove N-linked oligosaccharides by incubation at 37°C in G7 Reaction Buffer (NEB) supplemented with 1% NP40 for 48 h. Mock-treated antigens were obtained by the same procedure without addition of enzymes.

To digest proteins, SjEA or pSjEA was treated by proteinase K (Tiangen Biotech, China) at 56°C overnight, followed by heat inactivation of enzymes at 100°C for 10 min. In order to fully hydrolyze peptide, carboxypeptidase Y (Sigma) was subsequently used to digest proteinase K-treated SjEA or proteinase K-treated pSjEA at 25°C overnight with heat inactivation. Mock treatment on SjEA or pSjEA was also carried out by the same procedure without addition of enzymes. Protein disruption was regularly checked by SDS-PAGE viewed by silver staining.

### Generation of monoclonal IgGs against SjEA

Mice were injected subcutaneously with 100 μg SjEA formulated in CFA for primary immunization followed by boosting immunization twice with 50 μg SjEA formulated in IFA with 2 weeks interval. Hybridomas by fusing B cells from splenocytes with myeloma cells were made following standard procedures by Abgent (China), and 3 hybridoma cell lines were obtained based on their stable growth and on high IgG binding activities with SjEA.

Large quantity of mAbs was generated from ascitic fluids in Balb/c mice that were injected with 200 μl IFA intraperitoneally 1wk before 5 × 10^6^ monoclonal hybridoma cells were injected into peritoneal cavity. Ascites began to be harvested around 10th day after injection of cells. The IgGs in collected ascites were affinity purified and enriched through protein G-Sepharose column (Pointbio, China). The purity of mAb was determined by SDS-PAGE by sliver staining; the antibody titer was determined by its reactivity with SjEA by ELISA, and anti-glycan nature of IgG antibodies was determined by competitive ELISA.

### Measurement of anti-glycan IgGs in sera and in monoclonal antibodies with ELISA

Levels of anti-SjEA IgG was determined as described by ELISA [12]; whereas detection of anti-pSjEA IgG antibodies was performed with periodate treatment of egg antigens being carried out in plates as described [10]. Plates were coated overnight with 5 μg/ml of SjEA in carbonate-bicarbonate buffer (pH9.6). After being washed twice with 0.05 M sodium acetate (pH 4.6), 100 μl solutions containing 10 mM sodium-periodate dissolved in 0.05 M sodium acetate or containing 0.05 M sodium acetate were added to SjEA coated in wells for 1 h at 37°C to make pSjEA. After washing plates with sodium acetate twice and with PBS once, the plates were treated with 50 mM sodium borohydride in PBS for 30 min at room temperature to stop periodate reaction. Mock-treated SjEA was subjected to the same procedure without sodium-periodate treatment. Plates were then extensively washed by PBS containing 0.05% Tween-20 (PBST) for 5 times and blocked with PBST containing 3% BSA for 1 h at 37°C. Sera or monoclonal antibodies were added at appropriate dilutions and incubated for 2 h at 37°C. After plates were washed with PBST, HRP-labeled goat anti-mouse IgG (H + L) was added at 1:10000 and incubated for another 1 h at 37°C. Final color development was achieved by addition of ABTS (2, 2′-Azino-bis (3-Ethylbenzthiazoline-6-Sulfonic Acid) (Sigma) with absorbance at 405 nm by plate reader (Thermo scientific) after plates being extensively washed. Endpoint titers are defined as the lowest antibodies dilution which gives rise to an average OD that is 2.1 times greater than that of PBS.

The sera from infected mice were collected six weeks after *Schistosoma japonicum* infection. Human sera from 7 normal subjects and 7 patients who were positive for eggs in the feces by Kato-Katz test were kindly provided by the Institute of Infectious Disease and Vaccine Development, Tongji University. The detection was made by HRP-conjugated anti-human IgG (Promega) diluted at 1:10000.

Anti-glycan nature of IgG antibodies was determined by competitive inhibition assay through ELISA by proteinase K and carboxypeptidase Y-treated antigens. Sera or monoclonal antibodies with appropriate dilution were mixed first with serially diluted various kinds of antigens including mock SjEA or pSjEA, proteinase K-treated SjEA or pSjEA, proteinase K and carboxypeptidase Y-treated SjEA or pSjEA and OVA as irrelevant protein control for 2 h at room temperature before they were added onto SjEA- or pSjEA-coated plates. After incubation for 1 h at 37°C, protocol for ELISA described above was followed. Anti-glycan nature is indicated if the binding of IgGs with SjEA or pSjEA can be competed away by prior incubation with enzyme-treated SjEA or pSjEA. Percent inhibition due to presence of pretreatment with the maximal dose of treated egg antigens was calculated as following: percent inhibition = (OD without pretreatment − OD with pretreatment) / (OD 405 without pretreatment − OD blank) × 100.

### Immunofluorescence staining by monoclonal antibodies on worms

Monoclonal antibody was diluted in PBST containing 5% (w/v) BSA. 18 days old young worms and fresh cercariae of *Schistosoma japonicum* were fixed by 4% paraformaldehyde for 2 h. After being blocked with 5% BSA in PBS for 1 h at room temperature and washed three times with PBST, whole-mount cercariae or young worms were incubated with monoclonal antibody diluted at 2 μg/ml overnight at 4°C. After being washed in PBST, FITC-conjugated goat anti-mouse IgG (H + L) (1:400, KPL) was added for 30 min at room temperature in the dark. The slides were mounted with Antifade Mounting Medium (Beyotime Institute of Biotechnology, China). Images were acquired on confocal laser scanning microscope (Leica). Normal mouse IgG was used as negative control for staining.

### Protective immunity by passively transferred monoclonal IgGs

Female Balb/c mice were challenged with 30 ± 2 *Schistosoma japonicum* cercariae via abdominal skin penetration. One day before and 14 days after infection, 100 μg monoclonal antibodies were administered intraperitoneally. Protective immunity was evaluated based on worm and egg burdens as described [14,15]. Briefly, 42 days after challenge, all mice were euthanized. Both male and female adult worms were collected and counted by perfusing mice with cold PBS from mesenteric veins. Meanwhile, livers were isolated, weighed, minced and digested in 10 ml of 4% NaOH at 55°C for 2 h. Schistosome eggs released from infected livers were counted in 10 μl aliquots by light microscopic examination. Three aliquots were counted per liver sample and the average numbers were used as the egg burdens in each infected mice. Experiments were conducted for three times on different dates with different batches of cercariae and different batches of monoclonal antibodies generated.

### Statistical analysis

Unpaired Student’s *t* test and one-way ANOVA were used to determine statistical significance of differences among groups.

## Results

### IgGs from *Schistosoma japonicum* infected mice and patients demonstrated binding activities to both periodate-sensitive and periodate-resistant glycans

The IgG responses towards schistosome egg antigen-associated glycans were investigated on sera obtained from *Schistosoma japonicum* infected mice and humans via competitive ELISA assay using proteinase K and carboxypeptidase Y-treated egg antigens. As reported [9,10], IgGs from infected mice or humans were found to bind significantly less with periodate-treated egg antigen (pSjEA) than with mock-treated egg antigen (SjEA) by ELISA (Figure 1A and B). To determine whether glycans on egg antigens contribute to IgG binding reactivities with untreated SjEA, competitive ELISA assay is used to measure loss of IgG binding activities with egg antigens if optimally diluted sera is pre-incubated with antigens devoid of proteins by proteinase K and carboxypeptidase Y treatment. As shown in Figure 1C, comparing to inhibition by different doses of untreated SjEA, proteinase K-treated SjEA as well as proteinase K and carboxypeptidase Y-treated SjEA were able to inhibit IgGs binding activities with SjEA almost as much as untreated SjEA when infected human sera were examined. When similar inhibition assay was performed on IgGs from infected mice, about 50% reduction was consistently observed by proteinase K and carboxypeptidase Y-treated SjEA.

Competitive ELISA assay was then used to measure the remaining binding activities of IgGs towards pSjEA, we unexpectedly found that pSjEA treated by proteinases was still able to inhibit IgGs binding with pSjEA almost as much as untreated pSjEA both in infected mice and in infected humans (Figure 1D). This particular finding strongly indicated that periodate treatment in fact does not totally destroy glycan epitopes as believed [9,10]. Indeed, glycans positive for lectin WGA binding were still observed in periodate-treated SjEA, although glycans positive for ConA or for anti-Lewis^X^ antibodies were both diminished (Figure 1E). More importantly, it indicated that some of these periodate-resistant glycans actually contribute to antigenicity or immunogenicity of egg antigens for induction of IgG response in *Schistosoma japonicum* infection. It is both periodate-sensitive and periodate-resistant glycans that together confer glycan-associated dominant IgG responses seen in schistosome infection.

### Both periodate-sensitive and periodate-resistant glycan-specific monoclonal antibodies were successfully generated from SjEA immunized mice

**Figure 2:**
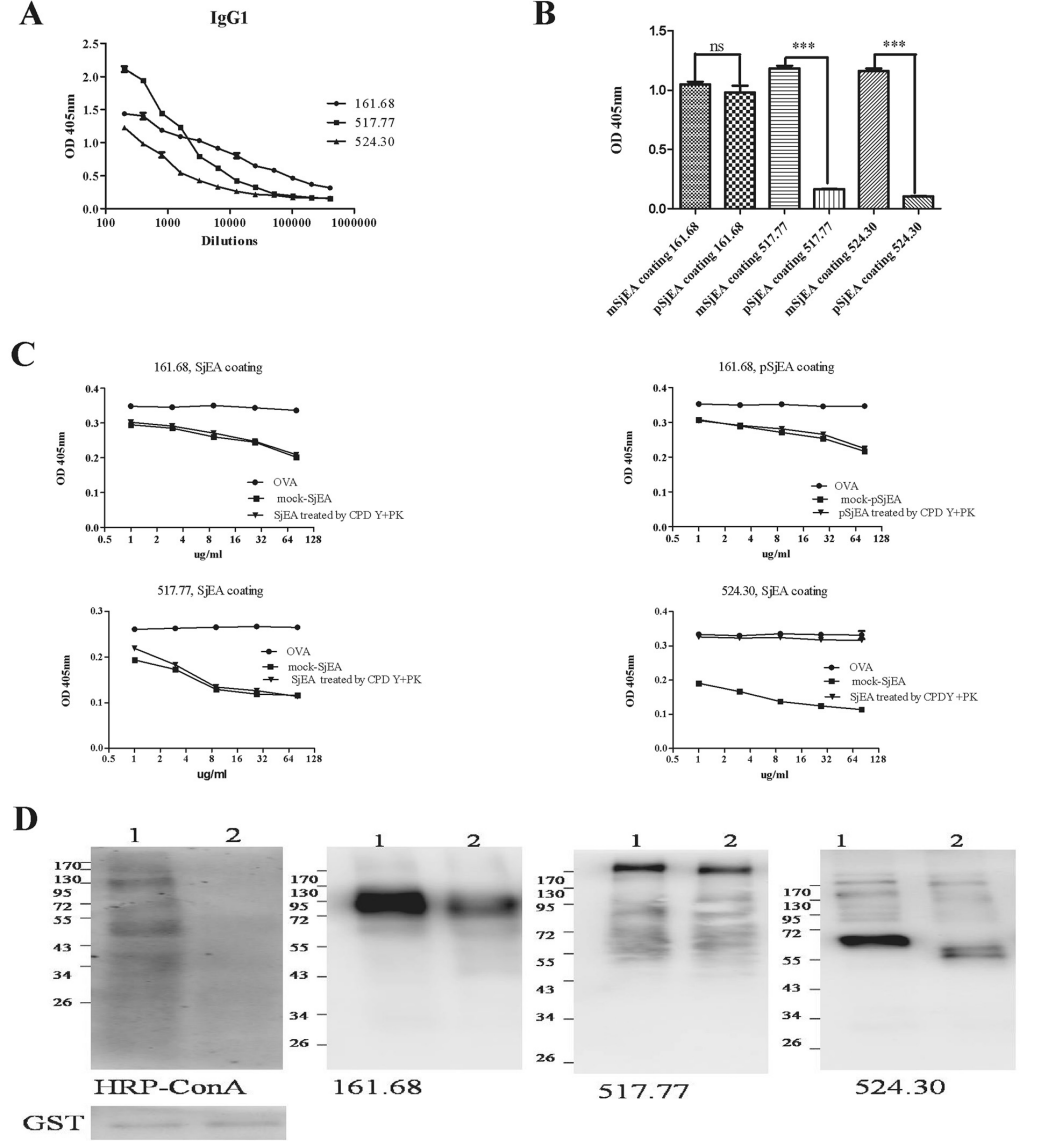
Characterization of anti-glycan natures on monoclonal antibodies. **A.** The bindings of monoclonal antibodies with SjEA were measured by ELISA and presented as mean ± SEM of the OD values from triplicate wells at different dilutions. **B.** Comparison of bindings of monoclonal IgGs with SjEA versus with pSjEA by ELISA. *t* test was performed on mean values obtained from triplicate wells from single experiment with *** as p < 0.001. **C.** Inhibitory effects on bindings of monoclonal antibodies by egg antigens devoid of proteins. Similar methods were applied as in Figure 1C and D. Data shown were mean ± SEM values from triplicate wells. **D.** Mock- treated (lane 1) or PNGase F-treated egg antigens (lane 2) were detected by monoclonal antibodies with western blot analysis. HRP-ConA was used to test the effect of PNGase F digestion on SjEA and anti-GST was used as loading control. Shown was a representative of at least three separate experiments.

To better study glycan antigenicity in egg antigens, monoclonal antibodies were generated from Balb/c mice immunized with SjEA emulsified in complete freund’s adjuvant. We obtained three hybridoma clones which can stably secrete high levels of SjEA-specific IgGs with isotype defined as IgG1. The endpoint titer of IgGs from these 3 clones named as 161.68, 517.77 and 524.30 respectively was high to 409,600, 25,600 and 12,800 (Figure 2A).

The periodate-sensitive or periodate-resistant nature of glycan epitopes recognized by monoclonal antibodies were then determined by ELISA assay with egg antigens treated with periodate or enzymes. Recognition of SjEA by mAb 161.68 was resistant to periodate treatment (Figure 2B). Furthermore, the bindings between SjEA or pSjEA and mAb 161.68 were all counteracted at similar levels by proteinase K and carboxypeptidase Y-treated egg antigens compared to untreated egg antigens (Figure 2C). On the other hand, recognition of SjEA by mAb 517.77 and 524.30 was sensitive to periodate treatment (Figure 2B), proteinase K and carboxypeptidase Y-treated SjEA was only able to counteract the bindings between 517.77 and SjEA but not between 524.30 and SjEA (Figure 2C). Contribution of N-glycan to epitopes recognized by mAbs was directly assessed by PNGase F treatment, which has been applied to release N-glycans on egg antigens [8]. The effectiveness of enzyme treatment on SjEA was demonstrated on loss of ConA recognition [16] as shown in Figure 2D. After egg antigens were digested by PNGase F, markedly reduced levels of detection of SjEA were found when probed by 161.68 and 524.30 comparing to mock-treated SjEA. In contrast, no changes were found when probed by 517.77 (Figure 2D).

Therefore, monoclonal antibodies of 161.68 likely recognize periodate-resistant N-glycan-associated epitopes; monoclonal antibodies of 517.77 likely recognize periodate-sensitive glycan epitopes, and monoclonal antibodies of 524.30 may recognize protein-glycan-conjugated epitopes.

### Both periodate-sensitive and periodate-resistant glycan-specific monoclonal antibodies provide protective immunity towards *Schistosoma japonicum* infection in mice

**Figure 3:**
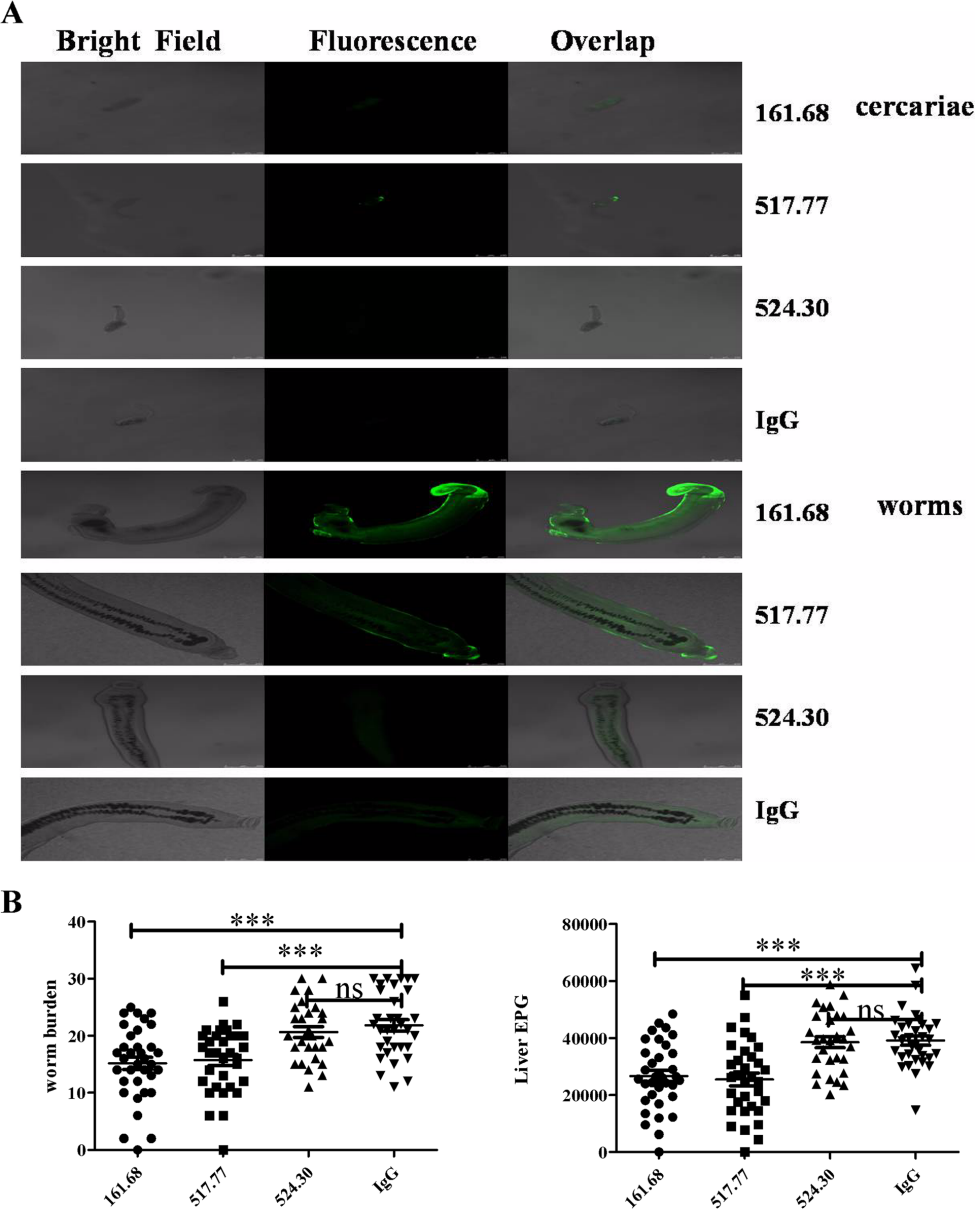
Anti-glycan monoclonal antibodies provide protective immunity towards *Schistosoma japonicum* infection in mice. **A.** Immunofluorescence localization of antigens recognized by mAbs on cercaria and young worm of *Schistosoma japonicum*. Normal mouse IgG was used as negative control. **B.** Combined results from 3 separate animal experiments were shown on worm and egg burdens in infected mice treated with mAbs of 161.68 (n = 35), 517.77 (n = 33), 524.30 (n = 29) and mouse IgG (n = 33). EPG stands for the number of eggs per gram of liver tissue. One-way ANOVA was used to analyze the significance with *** as p < 0.001.

Surface-exposed antigens on cercaria and schistosomula are commonly believed to be valid antigens to be targeted for establishment of effective antibody-mediated protective immunity against worm infection [17,18]. Immunofluorescence staining was performed to quest if these monoclonal antibodies towards egg antigen-associated glycans can also recognize glycan antigens on cercariae or young worms and their distributions. The results showed that positive immunostaining by 517.77 was found at the surfaces of cercariae as well as on surfaces of young worms. The positive staining by 161.68 was mainly distributed at the surfaces of young worms. 524.30 and mouse IgG, however, failed to demonstrate any significant staining on surfaces of cercariae or young worms examined (Figure 3A). Thus, monoclonal antibodies obtained against either periodate-sensitive or periodate-resistant glycans also recognize surface glycan antigens on cercariae or young worms which are cross-reactive with egg glycans.

The effects of anti-glycan IgG antibodies in protective immunity were assessed by administration of monoclonal antibodies prior to infection with mouse IgG as control. All 3 monoclonal antibodies and mouse IgG were individually given at 100 μg each time intraperitoneally 1 day before and 14 days after *Schistosoma japonicum* infection. Forty two days after infection, the number of worms recovered from mesenteric vessels and the number of eggs recovered from livers were counted and compared among different treatment groups. There were no significant differences among treatments by normal mouse IgG, by 524.30 and without treatment in terms of worm burden and egg numbers recovered (Additional file 1: Figure S1). However, treatment of mice with either 161.68 or 517.77 provided statistically significant protective responses to the infected mice, in that worm burden was reduced by 31.22% and 28.57% respectively and egg numbers in livers were reduced by 31.83% and 34.96% (Figure 3B). No protective effects were seen when doses of monoclonal antibodies given were reduced in half (Additional file 2: Figure S2). Furthermore, egg burdens were not disproportionately reduced compared with burdens of worms, suggesting parasite fecundity was not greatly affected. Thus, both anti-periodate-sensitive glycan antibodies and anti-periodate-resistant glycan antibodies can provide some level of protection to infected hosts.

## Discussion

Antigens treated by periodate oxidation have been widely accepted as deglycosylated antigens with loss of glycan-determined antigenicity. Such assumption has facilitated conclusions made by these studies [9,10] that IgGs elicited in schistosome infections are mainly against glycans. Our results are in agreement with the conclusions via more thoroughly investigated mechanisms. We used proteinase K-treated antigens in competitive ELISA in order to elucidate glycan contribution on antigenicity or immunogenicity. We found that predominantly occurred anti-SjEA IgG responses compared to anti-pSjEA IgG responses were largely due to IgG reactivities with antigenic periodate-sensitive glycans, especially so in infected humans. Moreover, residual anti-pSjEA IgG responses were almost derived from antigenic periodate-resistant glycans. Therefore, the dominant anti-glycan IgG responses found in schistosome infected mice as well as in infected humans are not simply resulted from reduced reactivities with periodate-treated antigens as believed [9,10], but rather a collective consequence from dominant antigenicity conferred upon by both periodate-sensitive and periodate-resistant glycans.

Different sugar moieties are reported to possess different sensitivities towards periodate treatment [11]. Fucose and sialic acid are mostly destroyed by periodate oxidation, whereas mannose, galactose and N-acetylgalactosamine are partially sensitive. Destruction of fucose in periodate-treated antigens indicates that fucose-conjugated glycoconjugates including both glycoproteins and glycolipids may contribute to dominant IgG responses towards periodate-sensitive glycans. Several mono- or multiple-fucosylated LDN (GalNAc (β1, 4) GlcNAc) variants are found to be highly targeted by IgGs from *Schistosoma mansoni* infected individuals [19]. These structures include FLDN (Fuc (α1-3) GalNAc (β1-4) GlcNAc), LDNF (GalNAc (β1- 4) (Fucα1-3) GlcNAcβ1) and FLDNF (Fuc (α1-3) GalNAc (β1-4) [Fuc (α1-3)] GlcNAcβ1). Considering LDNF can also be expressed by mammalian hosts [20-22], it is likely that fucose-containing elements FLDN and FLDNF may contribute more to antigenic periodate-sensitive epitopes in egg antigens. The other fucose-containing structures considered as periodate-sensitive antigens should be Lewis^X^ elements (Gal (β1-4) [Fuc (α1-3)] GlcNAc), particularly the multimeric forms of Lewis^X^ which are known to be expressed in schistosome but less in mammalian hosts [8,23,24]. In terms of periodate-resistant glycan epitopes, it is noteworthy to mention that N-acetylglucosamine is reported to be largely resistant to periodate oxidation [11]. Meanwhile, LDN (GalNAc (β1-4) GlcNAc) which contains N-acetylglucosamine without fucose has been repeatedly shown to be antigenic but not as much as its fucose derivatives in infected animals and humans [16,19,25]. Although like LDNF, LDN is also found to be expressed by mammalian hosts [20-22], we cannot exclude the possibility that LDN can contribute to periodate-resistant glycans-associated antigenicity demonstrated in present study.

It has been a long sought-after answer whether anti-glycan IgG or IgE responses developed in schistosome infection can be protective [26]. A rat IgG2a monoclonal antibody against an undefined 38 Kd *Schistosoma mansoni* surface antigen has been found to confer protective immunity when given before schistosome infection. Since the binding of this particular monoclonal antibody to 38 Kd antigen can be competitively inhibited by presence of KLH but not deglycosyalted KLH, thus it has been concluded that anti-glycan IgG may provide protective immunity [27,28]. Our results, however, more directly demonstrated that anti-glycan IgG responses, whether towards periodate-sensitive glycans or towards periodate-resistant glycans, can be protective.

It is intriguing then that schistosome infection always progresses even though high levels of anti-glycan IgG responses are seen during natural infection or egg vaccination [9,29]. Many factors may attribute to lack of apparent protective immunity in natural infection with ongoing dominant anti-glycan IgG responses. Firstly, the anti-glycan IgGs induced in natural schistosome infections are not represented or under-represented by relevant protective glycan epitopes. Two different patterns are detected by protective monoclonal antibodies in present study. The antigen recognized by 161.68 was about 95 Kd in size; whereas 517.77 recognizes many bands including a prominent band above 170 Kd (Figure 2 and Additional file 3: Figure S3). When we attempted to reprobe by infected sera the protective epitopes in egg antigens immunoprecipitated by protective anti-glycan monoclonal antibodies 161.68, no bindings were detected (Additional file 3: Figure S3, A1 vs C1). Therefore the protective epitope recognized by 161.68 is not likely the immunodominant epitope occurred in natural infection. Secondly, the level of IgGs against protective epitope is not high enough. This cause can be seen when molecules pulled down by 517.77 were reprobed with infected sera. The binding intensities on the antigen above 170 Kd were markedly reduced (Additional file 3: Figure S3, B2 vs C2). Furthermore, a dose-effect relationship was observed that protective effects from anti-glycan mAbs were reduced when the amount of mAb administered was reduced to half (Additional file 2: Figure S2). Thus, we believe that protective immunity found by direct anti-glycan antibodies transfer prior to worm infection in this study may reflect an amplification of the protective immune response determined by protective glycan epitopes which is induced at insufficient levels during natural infection or egg vaccination.

It is not clear how anti-glycan antibodies mediate reduction of adult worms hence egg numbers. Complement- or antibody-dependent cell-mediated cytotoxicity of schistosomula may be important pathways to reduce worm burdens found in this study, since both anti-glycan monoclonal antibodies selected are able to recognize surface antigens on young worms although they were originally generated against egg antigens. Indeed, a monoclonal antibody to LDN is reported to be capable of efficiently killing schistosomula *in vitro* via activation of complement cascade [7]. Regardless of what the mechanisms are, the results presented in this study certainly guarantee important roles played by some glycan epitopes in conferring protective immunity against schistosome infection, and probably against worm infection in general. Anti-glycan response should not be considered solely as a smoke screen [26]. This may become more critical when we found that anti-glycan IgG responses wane during late stage of infection [Additional file 4: Figure S4], similar as reported [9,16]. Therefore, protective anti-glycan IgG responses, if there is any, appear to mainly function at early stage of schistosome infection. All together, it strongly points to the importance to include worm-specific protective glycan epitopes in vaccine candidates instead of simply using polypeptides generated by prokaryotic system.

## Conclusions

In summary, present study reported both periodate-sensitive glycans and periodate- resistant glycans are antigenic in *Schistosoma japonicum* infection. More importantly, we demonstrated that both kinds of anti-glycan responses can be protective using the monoclonal antibodies generated. Further identification of the molecular basis of protective epitopes will facilitate our understanding on mechanisms between host-worm interplay and on mechanisms of protective immunity induced by glycans. All these will provide novel targets for vaccines against schistosome infection.

## Additional files

Additional file 1: Figure S1. Worm and egg burdens of infected mice treated by normal mouse IgG (n = 10), 524.30 (n = 10) and untreated (n = 10). One-way ANOVA was used to analyze the significance with ns as p >0.05. Additional file 2: Figure S2. Worm burdens of mice passively administered with mAbs of 161.68 (n = 9) and 517.77 (n = 9) at 50 μg and control IgG at 100 μg (n = 10). One-way ANOVA was used to analyze the significance with ns as p >0.05. Additional file 3: Figure S3. Different patterns between sera from infected mice and protective monoclonal antibodies to recognize the antigens immunoprecipitated by 161.68 and 517.77. Relevant antigens from SjEA co-immunoprecipitated with mAbs 161.68 (lane 1) and 517.77 (lane 2) were reprobed by 161.68 mAbs (A), 517.77mAbs (B) and sera from infected mice (C). Sizes shown are in kDa. Additional file 4: Figure S4. ELISA analysis of anti-peptide nature of IgGs in sera from 11w infected mice. The levels of anti-SjEA and anti-pSjEA IgG in sera from *Schistosoma japonicum* infected mice on 77 day (A). Differently treated SjEA or pSjEA competes IgG bindings with SjEA (B) or with pSjEA (C) in sera from 11w infected mice. ELISA data shown is one representative animal from 3 independent mice.

## Abbreviations

SjEA: Soluble *Schistosoma japonicum* egg antigens
pSjEA: Periodate treated- soluble *Schistosoma japonicum* egg antigens
