# Supplementary figures and images for: Protective immunity against Schistosoma japonicum infection can be provided by IgG antibodies towards periodate-sensitive or periodate-resistant glycans
Source: Parasit Vectors. 2015 Apr 18;8:234. doi: 10.1186/s13071-015-0842-1 (PMC4408597; doi:10.1186/s13071-015-0842-1)

**A**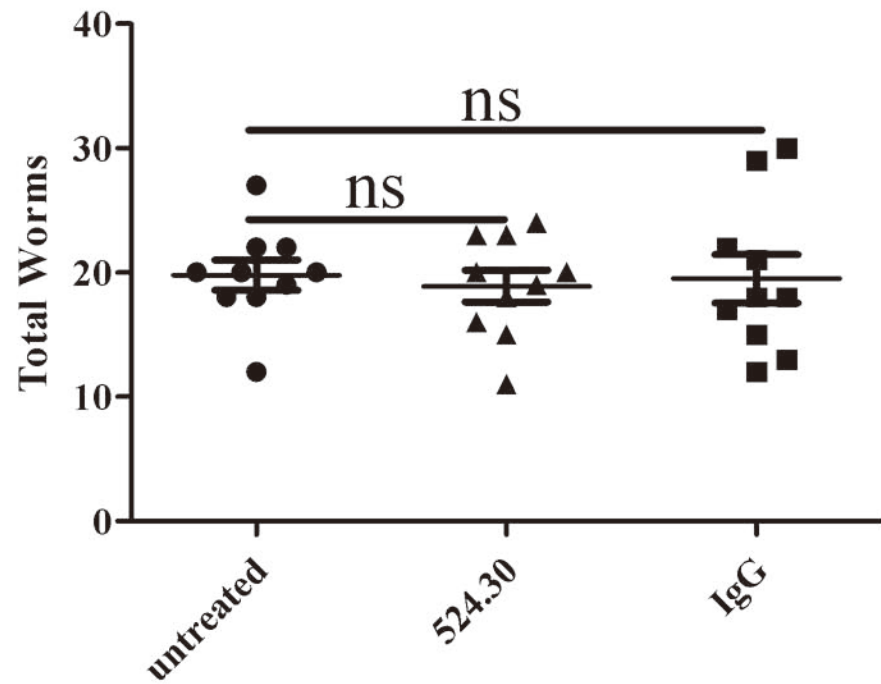**B**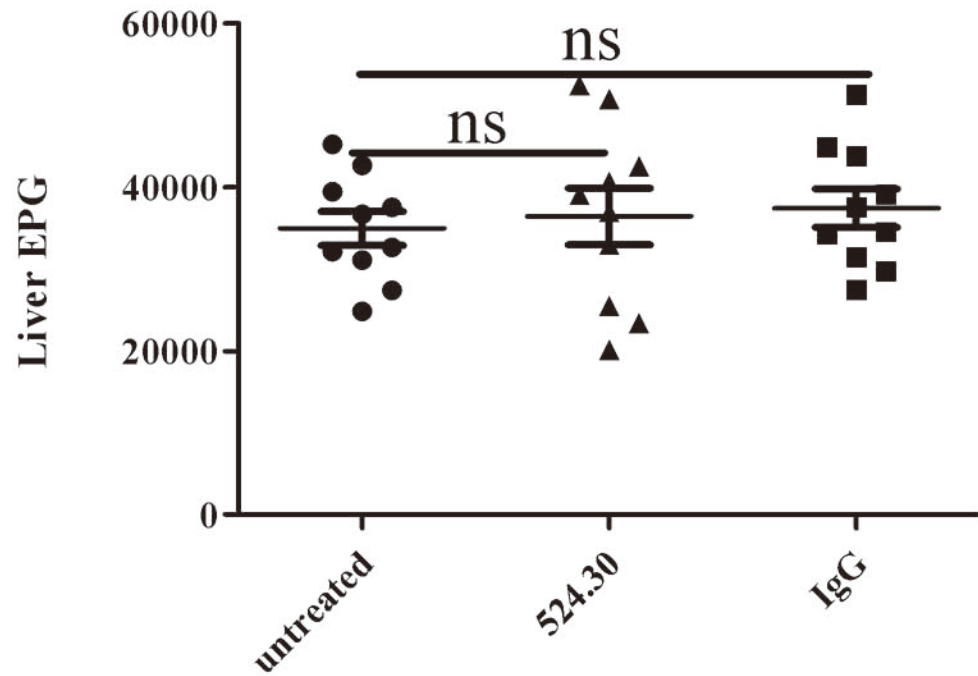

Supplement: Additional file 1: Figure S1. — Worm and egg burdens of infected mice treated by normal mouse IgG (n = 10), 524.30 (n = 10) and untreated (n = 10). One-way ANOVA was used to analyze the significance with ns as p >0.05. [file 13071_2015_842_MOESM1_ESM.pdf]

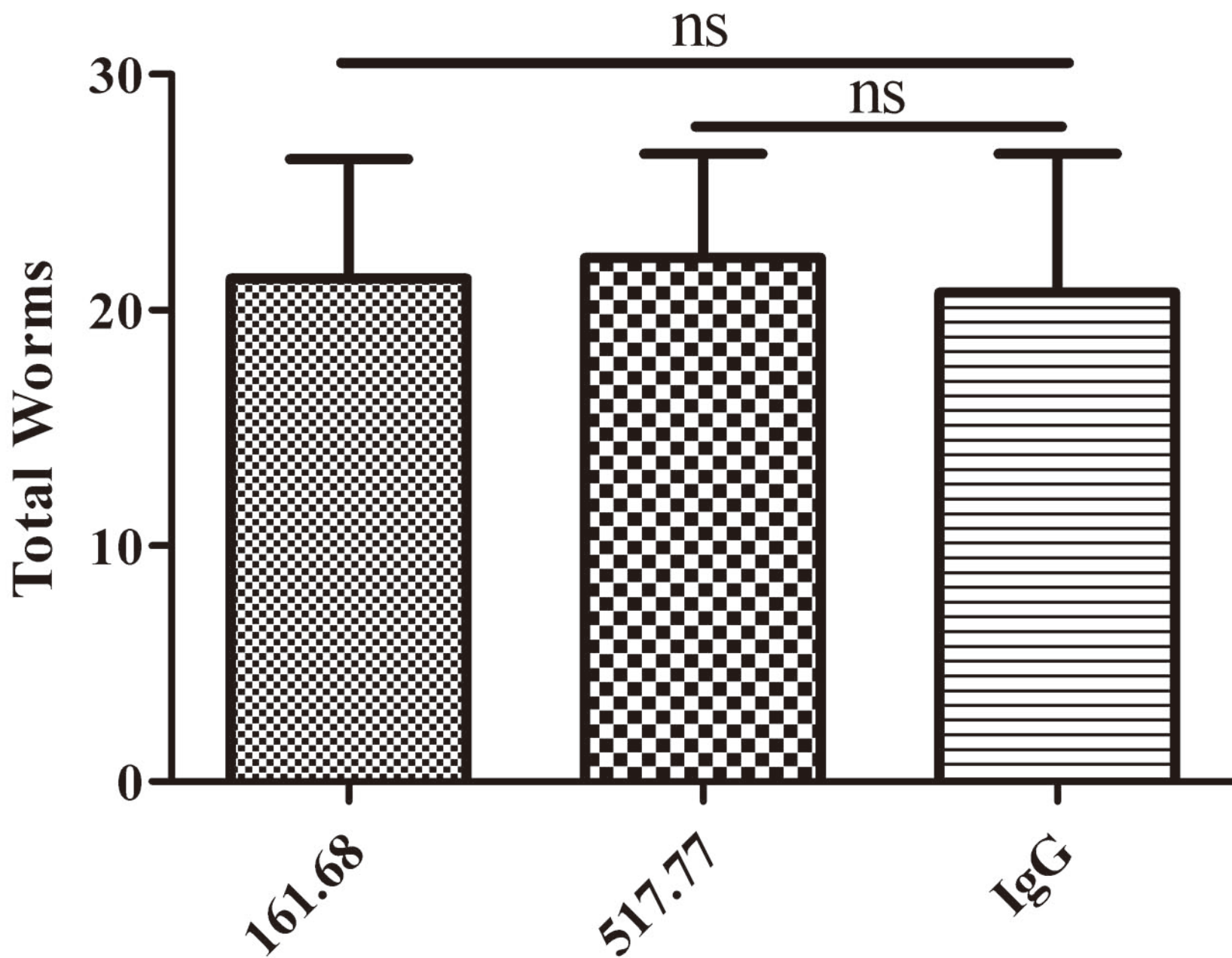

Supplement: Additional file 2: Figure S2. — Worm burdens of mice passively administered with mAbs of 161.68 (n = 9) and 517.77 (n = 9) at 50 μg and control IgG at 100 μg (n = 10). One-way ANOVA was used to analyze the significance with ns as p >0.05. [file 13071_2015_842_MOESM2_ESM.pdf]

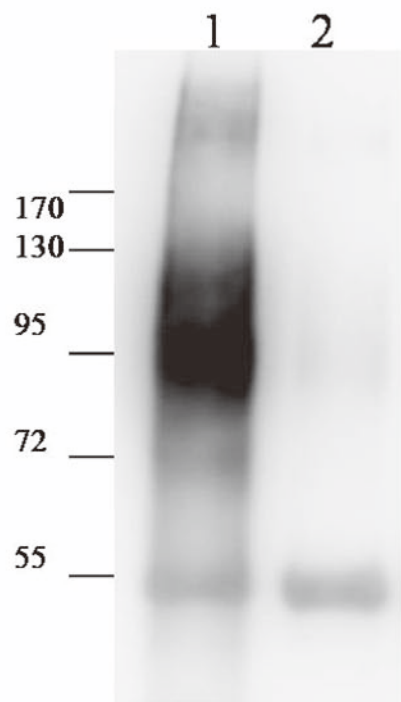

A

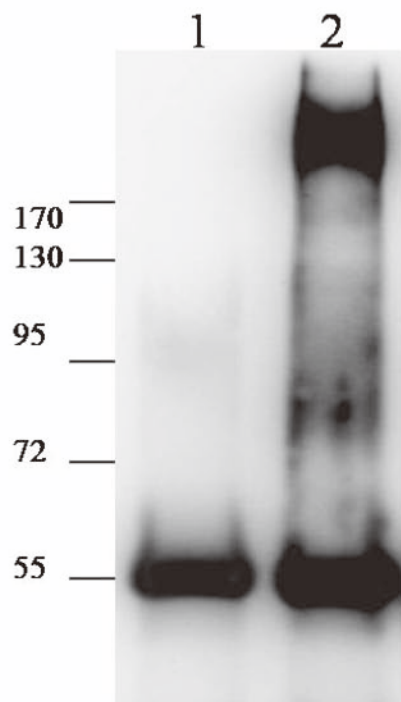

B

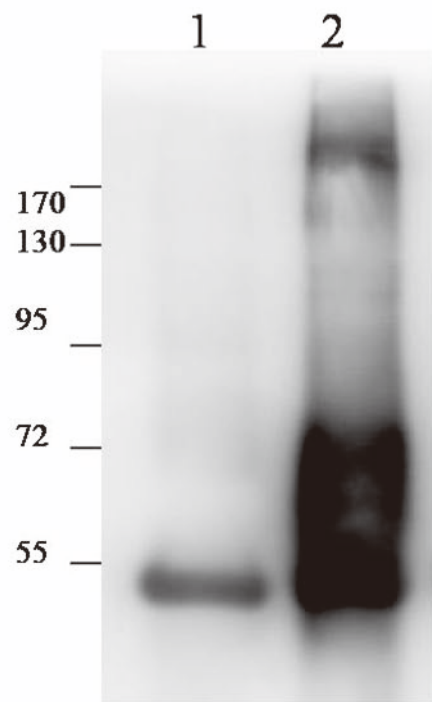

C

Supplement: Additional file 3: Figure S3. — Different patterns between sera from infected mice and protective monoclonal antibodies to recognize the antigens immunoprecipitated by 161.68 and 517.77. Relevant antigens from SjEA co-immunoprecipitated with mAbs 161.68 (lane 1) and 517.77 (lane 2) were reprobed by 161.68 mAbs (A), 517.77mAbs (B) and sera from infected mice (C). Sizes shown are in kDa. [file 13071_2015_842_MOESM3_ESM.pdf]

**A**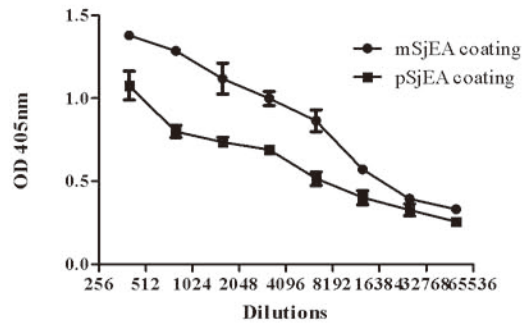**B**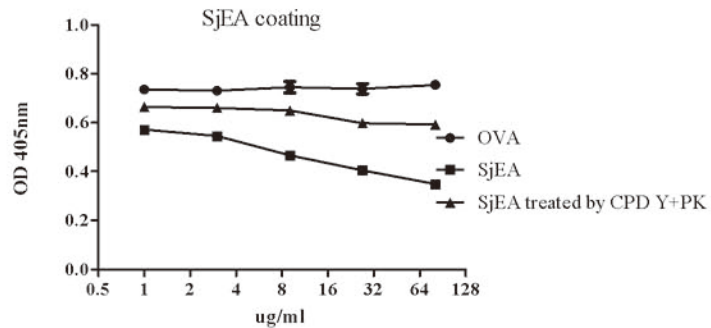**C**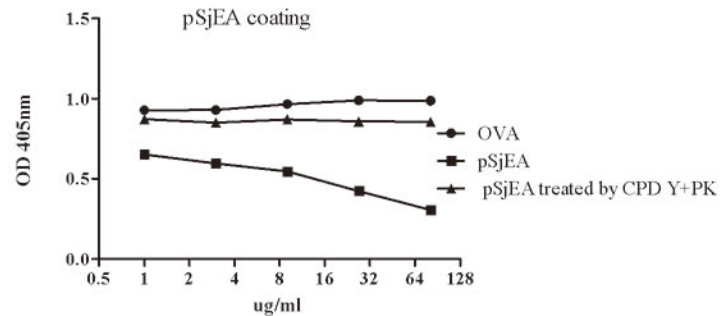

Supplement: Additional file 4: Figure S4. — ELISA analysis of anti-peptide nature of IgGs in sera from 11w infected mice. The levels of anti-SjEA and anti-pSjEA IgG in sera from Schistosoma japonicum infected mice on 77 day (A). Differently treated SjEA or pSjEA competes IgG bindings with SjEA (B) or with pSjEA (C) in sera from 11w infected mice. ELISA data shown is one representative animal from 3 independent mice. [file 13071_2015_842_MOESM4_ESM.pdf]
